# Oncogenic Ras is downregulated by ARHI and induces autophagy by Ras/AKT/mTOR pathway in glioblastoma

**DOI:** 10.1186/s12885-019-5643-z

**Published:** 2019-05-14

**Authors:** Chen Zhong, Mengting Shu, Junyi Ye, Xiaoxiong Wang, Xin Chen, Zhendong Liu, Wenyang Zhao, Boxian Zhao, Zhixing Zheng, Zhiqin Yin, Ming Gao, Haiqi Zhao, Kaikai Wang, Shiguang Zhao

**Affiliations:** 1 Department of Neurosurgery, The First Affiliated Hospital of Harbin Medical University, No. 23 Youzheng Street, Nangang District, Harbin, 150001 Heilongjiang Province People’s Republic of China; 2 Institute of Brain Science, Harbin Medical University, No. 23 Youzheng Street, Nangang District, Harbin, 150001 Heilongjiang Province People’s Republic of China; 3 Institute of Neuroscience, Sino-Russian Medical Research Center, Harbin Medical University, No. 23 Youzheng Street, Nangang District, Harbin, 150001 Heilongjiang Province People’s Republic of China; 4 Department of Pharmacology, The State-Province Key Laboratories of Biomedicine-Pharmaceutics of China, College of Pharmacy of Harbin Medical University, No. 157 Baojian Street, Nangang District, Harbin, 150001 Heilongjiang Province People’s Republic of China

**Keywords:** ARHI, Autophagy, Apoptosis, Glioblastoma, Ras

## Abstract

**Background:**

Glioblastoma is a disease with high heterogeneity that has long been difficult for doctors to identify and treat. ARHI is a remarkable tumor suppressor gene in human ovarian cancer and many other cancers. We found over-expression of ARHI can also inhibit cancer cell proliferation, decrease tumorigenicity, and induce autophagic cell death in human glioma and inhibition of the late stage of autophagy can further enhance the antitumor effect of ARHI through inducing apoptosis in vitro or vivo.

**Methods:**

Using MTT assay to detect cell viability. The colony formation assay was used to measure single cell clonogenicity. Autophagy associated morphological changes were tested by transmission electron microscopy. Flow cytometry and TUNEL staining were used to measure the apoptosis rate. Autophagy inhibitor chloroquine (CQ) was used to study the effects of inhibition at late stage of autophagy on ARHI-induced autophagy and apoptosis. Protein expression were detected by Western blot, immunofluorescence and immunohistochemical analyses. LN229-derived xenografts were established to observe the effect of ARHI in vivo.

**Results:**

ARHI induced autophagic death in glioma cells, and blocking late-stage autophagy markedly enhanced the antiproliferative activites of ARHI. In our research, we observed the inhibition of RAS-AKT-mTOR signaling in ARHI-glioma cells and blockade of autophagy flux at late stage by CQ enhanced the cytotoxicity of ARHI, caused accumulation of autophagic vacuoles and robust apoptosis. As a result, the inhibition of RAS augmented autophagy of glioma cells.

**Conclusion:**

ARHI may also be a functional tumor suppressor in glioma. And chloroquine (CQ) used as an auxiliary medicine in glioma chemotherapy can enhance the antitumor effect of ARHI, and this study provides a novel mechanistic basis and strategy for glioma therapy.

**Electronic supplementary material:**

The online version of this article (10.1186/s12885-019-5643-z) contains supplementary material, which is available to authorized users.

## Background

Although great progress has been made in current medical science, cancer remains a principal enemy threatening human health and life. Glioma, which is associated with low median overall survival and a high rate of occurrence in brain tumors, is a disease that needs to be urgently addressed [1]. New and effective therapeutic targets should be promptly identified. Autophagy is a highly conserved catabolic process by which cells can recycle organelles and long-lived intracellular proteins [2]. Depending upon the cellular microenvironment, the induction of autophagy can either protect or kill metabolically active cancer cells [3]. In the short term, autophagy can sustain cancer cells with multiple cellular stressors [4]. However, dysregulated or excessive autophagy could cause autophagic cell death, the type II programmed cell death. Aplasia Ras homolog member I (ARHI) is a powerful tumor suppressor gene belonging to the Ras superfamily located on human chromosome 1p31.3 and includes one promoter, two exons and one intron with a 687 bp protein-coding region that encodes a 26 kDa protein with 50–60% homology to Ras and Rap [5, 6]. However, the features of ARHI are totally different from those of the oncogene Ras. ARHI is downregulated in multiple malignant tumors, including ovarian cancer, breast cancer, lung cancer, prostate cancer, thyroid cancer, pancreatic cancer and glioma [7], and over-expression of ARHI at physiological levels can retard proliferation, reduce motility and enhance cancer cell dormancy [8]. ARHI has been recently reported to induce autophagy in ovarian and breast cancer cells and ARHI over-expression can decrease tumor growth, migration, and invasion in human glioma [9, 10]. But whether over-expression of ARHI can induce autophagy in human glioma cells and promote autophagic death are still unknown. Inhibition of autophagic function with chloroquine (CQ) can induce cancer death through necroptosis [11]. However, whether ARHI regulates autophagy in glioma and the relationship between this autophagy and glioblastoma has not been reported. Factors regulating the formation of ARHI induced autophaghy in glioma cells are still not completely understood.

Human cancer cells grown as subcutaneous xenografts in immunodeficient mice represent one of the most frequently used in vivo models for drug target validation and preclinical drug testing in translational cancer research. These cells are often engineered for inducible expression/suppression of a given gene of interest to enable a more precise assessment of its functions. So, we built subcutaneous xenografts to test the result in vivo. In the present study, we found that ARHI can induce autophagy-mediated cell death in glioma cells, the therapeutic efficacy of ARHI on glioma growth was preliminary verified in vitro and in vivo. We also found that using CQ after over-expression of ARHI enhanced apoptosis in glioma cells. ARHI-associated autophagy in glioma was further studied at molecular mechanism level. We found that ARHI can negatively regulate oncogenic Ras and inhibit RAS-AKT-mTOR signaling in glioma cell. Thus, ARHI may serve as a tumor suppressor gene and can regulate autophagy in glioma.

## Methods

### Cell lines and reagents

Human glioblastoma cell lines (LN229, T98G, U87, U251) and human brain astrocyte cell line NHA were obtained and authenticated from the China Infrastructure of Cell Line Resource at July 2017. All cell lines were recently authenticated by STR analysis and tested for mycoplasma contamination. LN229 (CRL-2611), T98G (CRL-1690), and U87 (HTB-14) were distributed by ATCC. CQ was purchased from Sigma Aldrich (St. Louis, MO, USA). 3-MA were purchased from MedChem Express (MCE, USA).

### Patient tissue preparation

A total of 9 glioma and 3 normal brain samples were obtained from the First Affiliated Hospital of Harbin Medical University between 2012 and 2014. The patients’ clinical characteristics, such as age, gender, and WHO grade, were collected. For qRT-PCR and western blot analysis, tissues were immediately frozen in liquid nitrogen.

### Cell transfection

The ARHI plasmids and lentiviruses used for ARHI overexpression and the negative control plasmids and lentiviruses were purchased from Genechem Co., Ltd. (Shanghai, China). Before transfection, glioma cells were cultured in six-well plates at 5 × 10^4^ cells per well. Then, ARHI lentiviruses were introduced into glioma cells treated with 8 μg/ml polybrene (Genechem). Transfection effects were observed by a fluorescence microscope after 48 h. Puromycin was used to purify the infected cells.

### Real-time PCR

Total RNA in cells and tissues was extracted using Trizol reagent, and the concentration of these RNA samples was measured using a spectrophotometer (Thermo Scientific™ NanoDrop 2000c). The RNA specimens were reverse transcribed into cDNA using a PrimeScript RT reagent kit (ToYoBo). The primer sequences for ARHI were forward 5′-CATAAGTTCCCCATCGTGC-3′; and reverse 5′-GAACAGCTCCTGCACATTCA-3′, and those for GAPDH used as a standard were forward 5′-ACCACAGTCCATGCCATCAC-3′; and reverse 5′-TCCACCACCCTGTTGCTGTA-3′.

### Western blot

The tissues and cells were lysed by RIPA buffer (Beyotime Institute of Biotechnology, Beijing, China) containing phenylmethylsulfonyl fluoride (PMSF) and a phosphatase inhibitor. The equal amounts of lysates were separated by 8–12.5% SDS-PAGE gels and transferred onto PVDF membranes. Antibody SQSTM1 (#23214), LC3B(#3868), Ras (#8955), Cleaved Caspase3 (#9662), mTOR (#2972), AKT (#9272) and Beta actin (#3700) were purchased from Cell Signaling Technology, ARHI antibody (ab107051) was purchased from abcam.

### Cell proliferation and clone formation assay

A density of 5000 cells were planted into 96-well plates for each well and mixed 10 μl MTT at 72 h. After 24 h of transfection with the ARHI and control plasmid, cells (500 cells/well) were seeded into a six-well plates and cultured for two weeks. Then, 0.1% crystal violet was used to stain clones, and cells were photographed using a ChemiDocTM MP system (Bio-Rad, USA).

### TUNEL assay

Cells were plated on coverslips, and after treatment, cells were washed twice with phosphate-buffered saline (PBS). They were then fixed with 4% paraformaldehyde for 20 min, blocked with 5% goat serum at 37 °C for 30 min and then treated with 0.3% Triton X-100 for 10 min. Next, 50 μl TUNEL reaction mix (Wanleibio, WLA030a) was added to each sample, and cells were incubated for 60 min at 37 °C in the dark. They were then incubated with DAPI (Beyotime, C1005), and photographs were captured by using a FSX100 Bio Imaging Navigator system (Olympus, Japan).

### Transmission electron microscopy

GBM cells were seeded onto 6-well plates at a density of 1 × 10^5^ cells per well. After treatment, cells were harvested and fixed using 2.5% glutaraldehyde at 4 °C overnight. Then, the harvested cells were dehydrated with ethanol and acetone and fixed with 1% osmium tetroxide. Samples were observed and captured by using transmission electron microscope (Hitachi H-7650, Japan).

### Cell immunofluorescence

A total of 5 × 10^4^ cells were seeded onto 35-mm glass-bottomed dishes. After treatment, cells were harvested and fixed with 4% paraformaldehyde. Then, cells were treated with 0.1% Triton X-100 and blocked with 5% bovine serum albumin (BSA, BOSTER, AR0004). Cells were incubated with primary antibody at 4 °C overnight and then incubated with Alexa Flour 594 AffiniPure goat anti-rabbit IgG (ZSGB-BIO, ZF-0516) and DAPI (Beyotime, C1005).

### Flow cytometry detected cell apoptosis

After transfecting ARHI for 24 h, cells were seeded onto a six-well plate and fixed with 3-MA or CQ. After 48 h, cells were harvested and detected using an Annexin V-PE Apoptosis Detection Kit (BD Bioscience, 556,422). Then, samples were measured by an Accuri C5 flow cytometer (BD Bioscience).

### GFP-RFP-LC3 lentivirus transfection and fluorescence imaging

Cells were transfected with GFP-RFP-LC3 lentivirus according to the manufacturer’s protocol. After treatment, the autophagosomes (yellow dots) and autolysosomes (red dots) were determined by augmented microscopy (BioTek Instruments, USA).

### Tumor xenograft model

All 28-day-old (15-20 g) BALB/C nude mice were purchased from the Vital River Animal Center (Beijing, China) randomly divided into four groups and each group have 6 mice. The required sample sizes were calculated and tabulated with different levels of accuracies and marginal errors with 95% confidence level for estimating and for various effect sizes with 80% power for purpose of testing as well. LN229 cells (5 × 10^6^ ARHI-LN229 cells was subcutaneously injected into the right hips of each mouse. In drug treatment group, the mice were injected with CQ (30 mg/kg/d) for 4 weeks. Tumor volume was assessed and calculated (volume = (width)^2^ × (length)/2) every 2 days. Mice were sacrificed on day 34, all mice had no panic, struggling, shouting. The procedure is as follows: After using Avertin (2, 2, 2-Tribromoethanol) anesthesia (the injection dose should reach lethal dose:> 0.5 mg/20 g, intraperitoneal injection), the heartbeat、breath and various reflexes of mice disappeared. No painful reaction was observed in the whole course of anesthesia. Then, mice were sacrificed by breaking neck for next step. After photography, tumor weight was measured. All experimental ethics and animal experiments were conformed to the European Parliament Directive (2010/63/EU) and were approved by the Institutional Animal Care and Use Committee at Harbin Medical University (No. HMUIRB-2008-06).

### Immunohistochemistry

Formalin-fixed and using paraffin embedded samples were sliced into 5-μm-thick sections. Then, sample sections were immunostained using primary antibodies Ki67, Ras, cleaved caspase-3 and ARHI at 4 °C overnight. Then, applying secondary antibodies at 37 °C for 30 min. Next, samples were visualized according to manufacturer’s protocol and using a diaminobenzidine (DAB) substrate kit for 10 min. After intensive washing, samples were counterstained with hematoxylin, dehydrated and coverslipped. Obtaining pictures by using FSX100 Bio Imaging Navigator (Olympus, Japan).

### Statistical analysis

Three independents experiments data are shown as the means ± standard deviations (SD). Two groups statistical analysis was performed using Student’s t test (two-tailed), *indicated a statistical significance of *P*-value< 0.05; **indicated a strong statistical significance of *P*-value< 0.01; *** indicated an even strong statistical significance of *P*-value< 0.001.

## Results

### ARHI has a lower expression level in glioma than in normal brain tissue and over-expression of ARHI can induce LN229 and T98G cells proliferation arrest

**Fig. 1:**
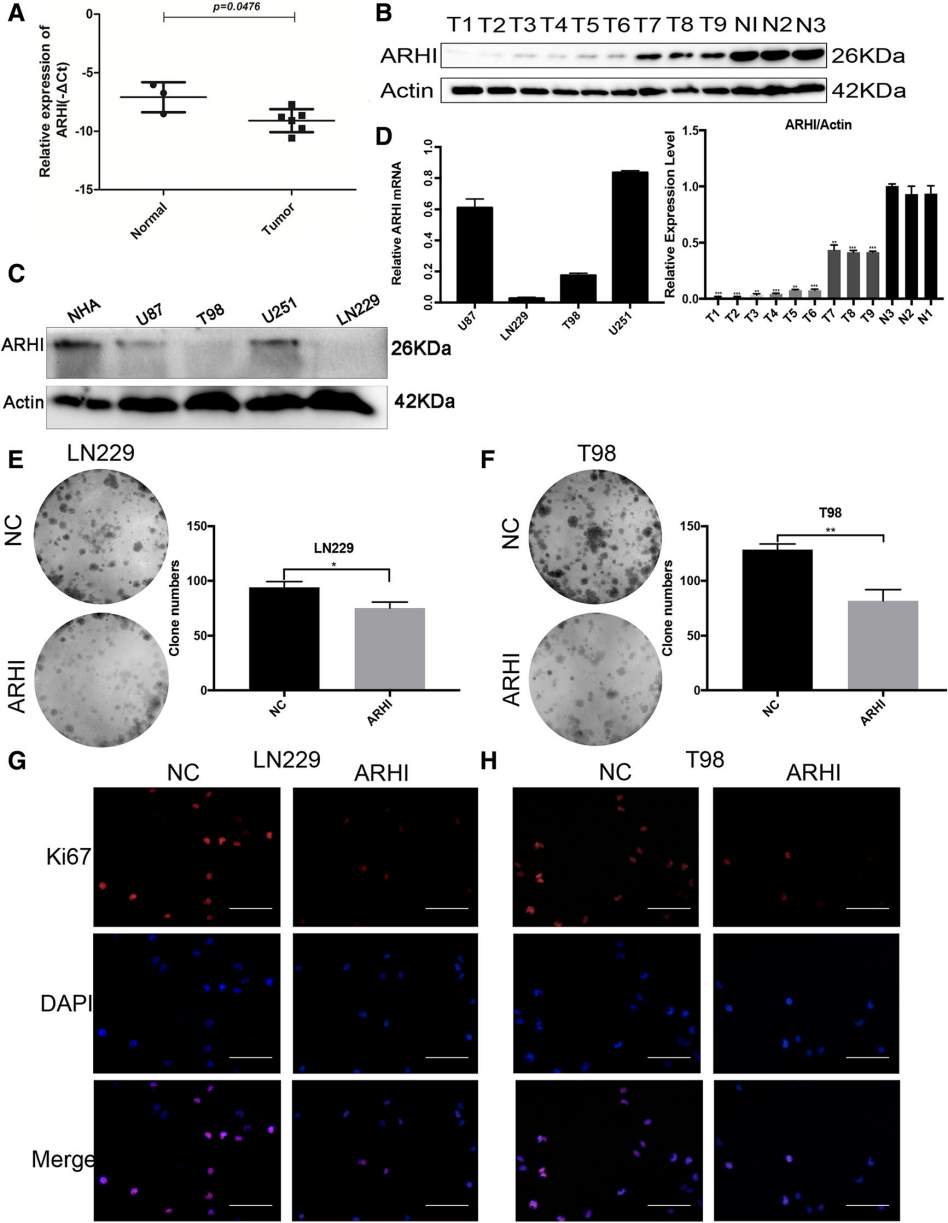
ARHI inhibits glioma cell growth in vitro. **a** ARHI mRNA relative expression in glioma and normal brain tissues. **b** ARHI expression in different grades of glioma tissues and normal brain tissues measured by western blot assays. T1-T3 grade IV; T4-T6 grade III; T7-T9 grade II; N1-N3 normal tissues. **c** ARHI expression in different glioma cell lines and human brain astrocyte cell line (NHA) (d) Relative ARHI mRNA expression in four different glioma cell lines. **e**-**f** Colony formation assays of LN229 and T98G cells after over-expressing ARHI contrast with the negative control. The experiments were repeated 3 times independently, and the bars represent SD. The data were normalized to the control group (*p < 0.05, **p < 0.01). **g**-**h** Fluorescence microscopy of Ki67 expression after transfection with ARHI and negative control plasmids. The scale bar represents 100 μm

We used RT-PCR and western blot to detect the expression levels in glioma tissues and normal brain tissue (Fig. 1a-b, Additional files 1 and 2). The results showed that glioma tissues had lower ARHI expression, and higher grades of glioma corresponded to lower ARHI protein expression (T1-T3 WHO IV, T4-T6 WHO III, T7-T9 WHO II, N1-N3 normal brain tissues). We also measured ARHI expression in the human glioma cell lines LN229, T98G, U251, and U87 and human brain astrocyte cell line NHA (Fig. 1c-d). Normal human brain astrocyte cell line had higher expression levels of ARHI than the four glioma cell lines. To examine the effect of ARHI on the viability of human glioma cells, we chose the lower expression cell lines LN229 and T98G to investigate the effect of over-expressing ARHI on glioma cell clonogenicity and proliferation. In Fig. 1e-f, colony formation was significantly decreased after over-expression of ARHI. We established stably infected LN229-ARHI and T98G-ARHI cells, cell proliferation was measured by Ki67 immunofluorescence dyeing. The proliferative marker Ki67 was decreased after over-expression of ARHI (Fig. 1g-h). These results indicate that ARHI induces cytotoxicity and inhibits cell growth in glioma cells and the similar phenomena were found in glioma stem cells (Additional file 3).

### Over-expression of ARHI can induce dysregulated autophagy in glioma cells

**Fig. 2:**
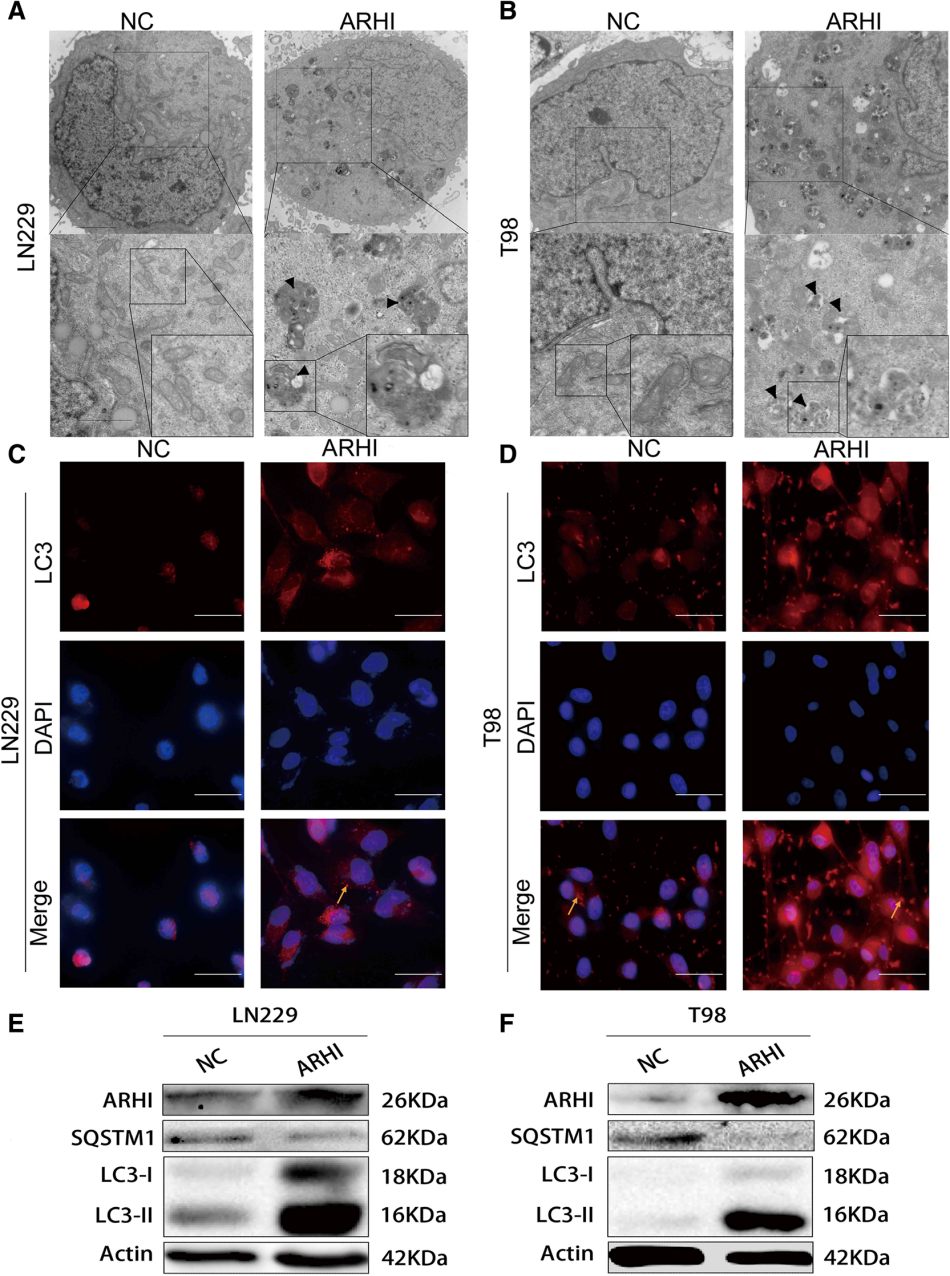
Over-expression of ARHI can induce autophagy in glioma cells. **a**-**b** Representative transmission electron microscopy of LN229 and T98G cells after transfection with ARHI and negative control plasmids. The scale bar represents 2 μm. **c**-**d** Fluorescence microscopy of LC3B expression after over-expressing ARHI and negative control plasmids (yellow arrows indicate LC3B puncta). The scale bar represents 50 μm. **e**-**f** After transfecting LN229 and T98G cells with ARHI and negative control plasmids, ARHI, SQSTM1 and LC3B expression was examined by western blot assay

Increasing evidence has shown that autophagy plays an important role in anticancer therapies [12]. ARHI has been reported to induce autophagy in many cancer cells, including ovarian cancer and breast cancer. Therefore, we questioned whether over-expression of ARHI can induce autophagy in glioma cells. After over-expressing ARHI, we used transmission electron microscopy to detect autophagic vacuoles. We found massive autophagosomes in the cell cytoplasm of the ARHI group compared with the negative control group (Fig. 2a-b), indicating that ARHI can induce autophagy in glioma cells. Abundant cytoplasmic protein LC3-I is an that is cleaved and lapidated and forming LC3-II, translocating to and associating with the autophagosome in a punctate pattern. Autophagy thus enable the cell to eliminate and recycle proteins or organelles to sustain metabolism and can be recognized in part by formation of LC3-II punctate. We using LC3B immunofluorescence stain to detect LC3B puncta (Fig. 2c-d) and over-expressing ARHI can dramatically increase LC3B puncta formation. SQSTM1 is widely used as a marker for autophagic degradation, as it is binding to LC3 and selectively degraded in autolysosomes. A smooth autophagy flux is associated with decreased levels of SQSTM1 [13]. Similarly, we found ARHI significantly induced autophagy with increased levels of LC3-II and decreased levels of SQSTM1. This western blot result showed that ARHI can enhance autophagy flux in glioma cells (Fig. 2e-f). In patients derived glioma cells, we got the same results (Additional file 4).

### Over-expression of ARHI can cause autophagic cell death and can reduce the levels of p-AKT and p-mTOR by negatively regulating Ras

**Fig. 3:**
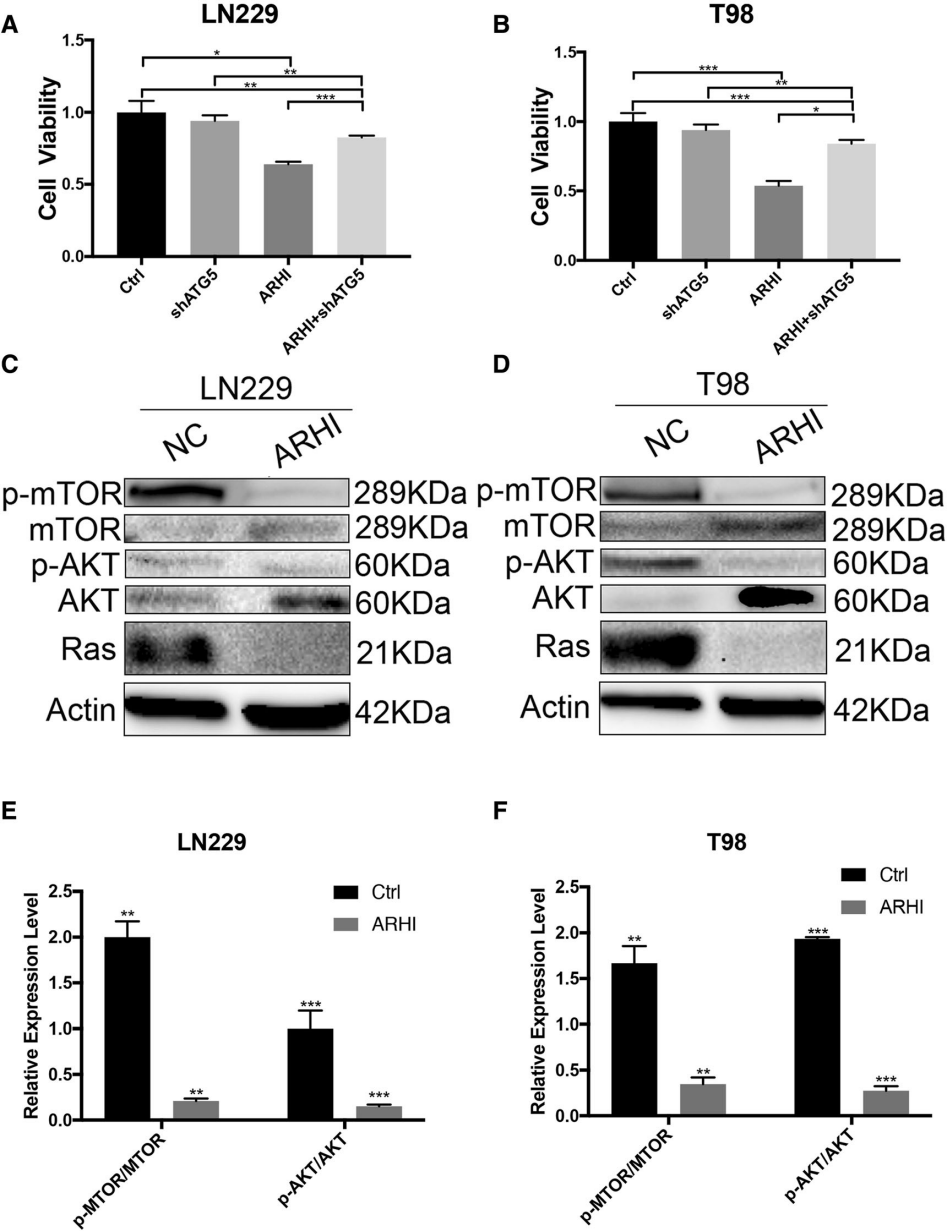
ARHI can induce autophagy-mediated glioma cell death by inhibiting Ras/AKT/mTOR signaling. **a**-**b** Cell viability of LN229 and T98G cells after over-expressing ARHI or inhibiting autophagy by shATG5 (the bar whiskers represent SD, *p < 0.05, **p < 0.01, ***p < 0.001). **c**-**d** After transfecting LN229 and T98G cells with ARHI and negative control plasmids for 72 h, the expression levels of phosphorylated and total mTOR/ AKT were assessed by western blotting. **e**-**f** Using total mTOR and AKT as the internal controls, the expression levels of phosphorylated mTOR and AKT were calculated (the bar whiskers represent SD, *p < 0.05, **p < 0.01, ***p < 0.001)

To investigate whether ARHI-induced glioma cell growth arrest depends on autophagy, we used shATG5 plasmids to infect LN229-ARHI and T98G-ARHI cells. ARHI expression significantly inhibited glioma cell growth within 72 h but exhibited little growth-inhibiting effect on ARHI-shATG5 cells (Fig. 3a-b), suggesting that ARHI induces autophagy-mediated glioma cell death. As ARHI is a member of the Ras superfamily, and it encodes a 26 KDa protein that has 50–60% homology to Ras [8]. Ras can regulate autophagy by indirectly enhancing AKT and down regulating mTOR phosphorylation levels. A previous study reported that ARHI induces autophagy in ovarian cancer cells by inhibiting Ras/MAP signaling [13]. Therefore, we questioned whether ARHI induces autophagy in glioma cells by regulating Ras. To further investigate the molecular mechanism of ARHI-associated autophagy in glioma, we used western blotting to detect Ras after over-expressing ARHI in glioma cells. We found that ARHI can decrease Ras expression and inbibit the activation of AKT and mTOR, then, promoting autophagy in glioma cells.

### CQ can cause excessive autophagy in ARHI-glioma cells

**Fig. 4:**
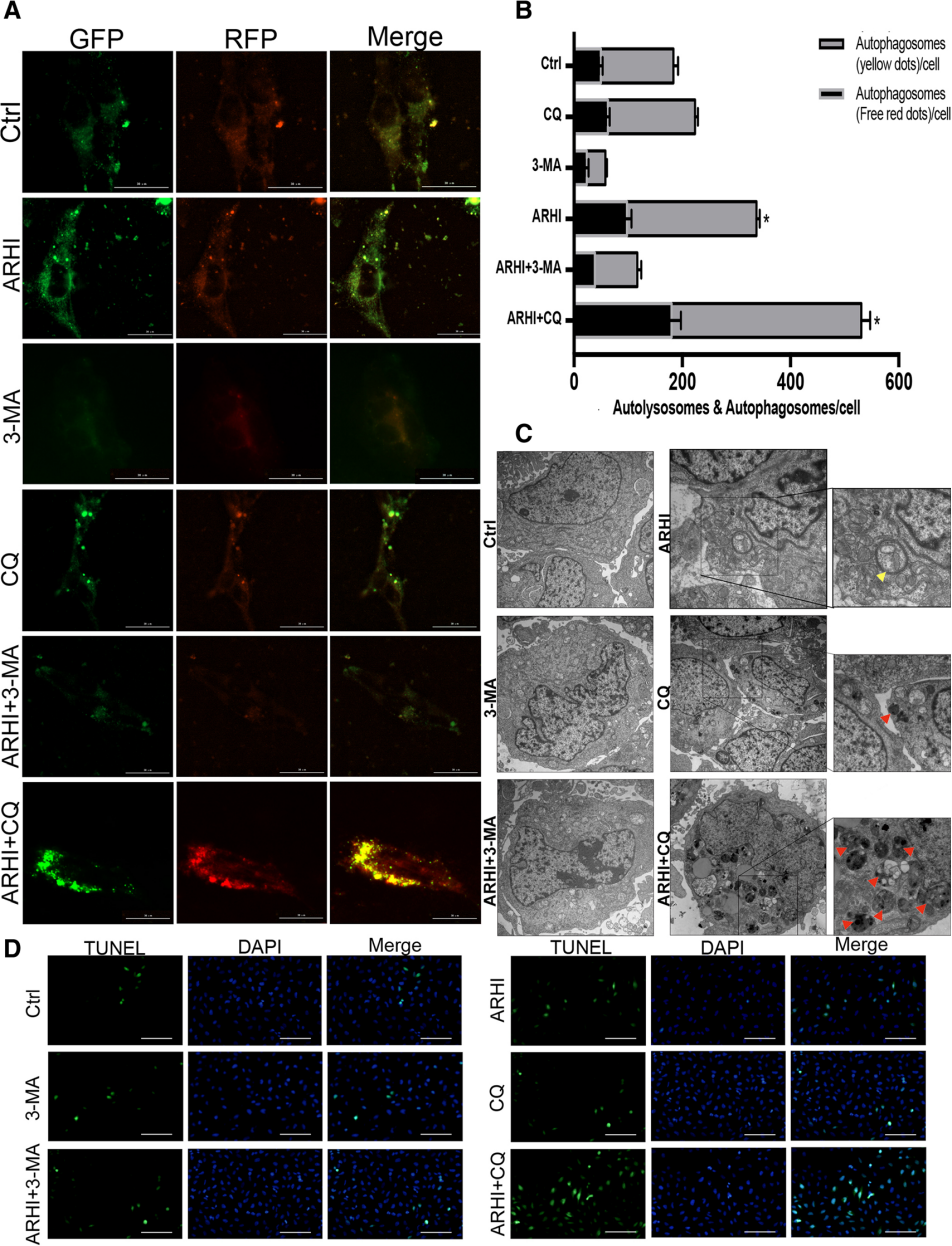
CQ can enhance the accumulation of autophagic vacuoles induced by ARHI in glioma cells. **a** The distribution of LN229 mRFP-GFP-LC3 immunofluorescence in cells transfected with ARHI and negative control plasmids cultured with 2 mM 3-MA or 3 μM CQ after 48 h was analyzed by fluorescence microscopy. Scale bar represents 30 μM. **b** The mRFP-GFP-LC3 expression levels in LN229 cells (the bar whiskers represent SD, *p < 0.05, **p < 0.01, ***p < 0.001). **c** TEM photomicrographs of LN229 cells. AVs, autophagic vacuoles; AVi, initial AVs (yellow arrows); AVd, late AVs (red arrows). The scale bar represents 2 μm. **d** Fluorescence microscopy of TUNEL expression in LN229 cells. The scale bar represents 100 μm

Recent reports have indicated that CQ can strengthen the autophagy inducer’s toxicity in cancer cells mainly because CQ can reduce/increase the apoptosis regulatory protein Bcl-2/cleaved caspase3, thus promoting apoptosis in cancer cells [14]. ARHI as an autophagy inducer, and it is unknown whether ARHI has synergistic action with CQ. Autophagic flux are monitored by GFP-RFP-LC3 fluorescence assays. The GFP signal can be digested in the lysosomes’ acidic conditions. Contrary to GFP, RFP can exist in acidic condition stably. Therefore, the stage of without lysosome fusion was indicated by the colocalization fluorescence of GFP and RFP, which suggests autophagosomes shown as yellow dots. In contrast, the RFP signal indicates autolysosomes shown as red dots [15, 16]. GFP-RFP-LC3 fluorescence assays showed that more yellow dots were aggregated in ARHI-LN229 cells after 48 h of exposure to CQ (3 μM). Studies have shown that 3-methyladenine (3-MA) can inhibit the formation of beclin1-PtdIns 3KC3 compounds, thus indirectly suppressing autophagosome formation. Thus, 3-MA can block the early stage of autophagy [17, 18]. In the 3-MA group (2 mM), the little yellow fluorescence was observed, and red dots were aggregated in ARHI-LN229 cells (Fig. 4a-b). This result indicated that CQ can inhibit the late stage of autophagy and that blockage of the late stage of autophagy is associated with increased levels of substrate proteins, including toxic proteins. 3-MA inhibited the early stage of autophagy but did not induce this effect. As we know, transmission electron microscopy (TEM) is the golden standard for detecting autophagosomes. In Fig. 4c, we use TEM for further observing autophagosomes. In ARHI-lN229 cells, initial autophagic vacuoles (AVi) were predominated and we found after adding CQ in ARHI-lN229 cells, more late autophagy vacuoles (AVd) were accumulated in the cytoplasm. Also, as the accumulation of AVd, and the accumulation of AVd may promote apoptosis in ARHI-lN229 cells. The morphological changes in CQ-treated ARHI-LN229 cells were examined with a TUNEL assay, and more apoptotic cells were detected in the CQ-treated ARHI-LN229 cells (Fig. 4d).

### Inhibition of autophagy at late stage can promote apoptosis in ARHI-LN229 cells through the excessive autophagy and reduction of anti-apoptotic protein Bcl-2

**Fig. 5:**
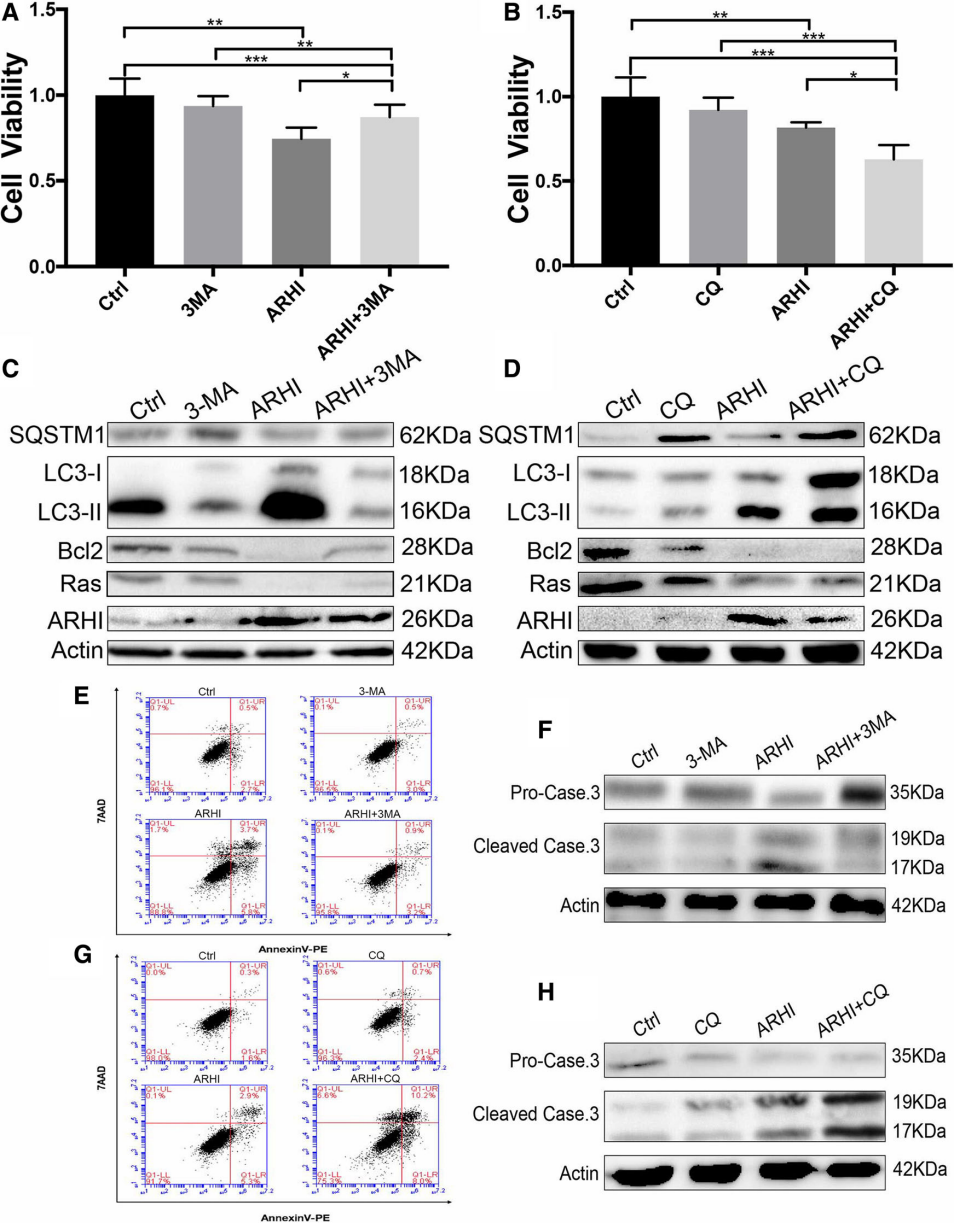
CQ can promote apoptosis in ARHI-glioma cells. **a**-**b** Cell viability of LN229 and T98G cells transfected with ARHI and negative control plasmids cultured with 2 mM 3-MA or 3 μM CQ after 48 h. **c**-**d** Western blot showing SQSTM1, LC3, Bcl2, Ras and ARHI levels in LN229 cells after treatment with 2 mM 3-MA or 3 μM CQ for 48 h. The experiments were repeated 3 times independently. **e**-**h** After treatment with 3-MA or CQ, LN229 cells were analyzed by flow cytometry and examined by western blot for apoptosis-related caspase-3 protein. All experiments were repeated 3 times independently

Inhibition of the late stage of autophagy can cause the accumulation of autophagic vacuoles and robust apoptosis [18–20]. In our research, we found that using CQ to inhibit late stage of autophagy can cause accumulation of autophagic vacuoles, increased the cytotoxicity of ARHI and raising apoptosis level in glioma cell. An MTT assay was used to determine the effect of different autophagy inhibitors, CQ and 3-MA, on the viability of the human glioma LN229 cells and ARHI-LN229 cells. As shown in Fig. 5a-b, the results indicated that 3-MA (2 mM) attenuated ARHI-induced autophagy-mediated glioma cell death, while CQ (3 μM) significantly enhanced the cytotoxicity of ARHI. In the western blot results, we found that either CQ or 3-MA could block the autophagic flux with increased levels of SQSTM1 (Fig. 5c-d). However, in the 3-MA groups, the reduced level of LC3-II and a reduced number of autophagic vacuoles indicated that 3-MA blocked ARHI-induced autophagy. Contrary to 3-MA, CQ treatment led to an increase in the level of LC3-II and the accumulation of autophagic vacuoles induced by ARHI. These results suggested that CQ enhanced the cytotoxicity induced by ARHI, partly because of the accumulation of autophagic vacuoles [21–23]. The accumulation of autophagic vacuoles can promote cancer cell death and apoptosis [24–28]. Thus, we questioned whether CQ enhanced the cytotoxicity of ARHI in glioma cells partly by promoting apoptosis. Furthermore, we evaluated cleaved caspase-3 and Bcl-2 protein expression levels and the percentage of apoptotic cells. The results showed that both the expression of apoptosis-related cleaved caspase-3 protein and the percentage of Annexin V-positive cells were increased in the CQ-treated ARHI-LN229 group (Fig. 5e-h). However, after treatment with 3-MA, the apoptotic levels in ARHI-LN229 cells were decreased. Taken together, CQ can promote ARHI-LN229 cell death partly by increasing its apoptosis levels.

### CQ enhances the cancer suppression of ARHI in vivo

**Fig. 6:**
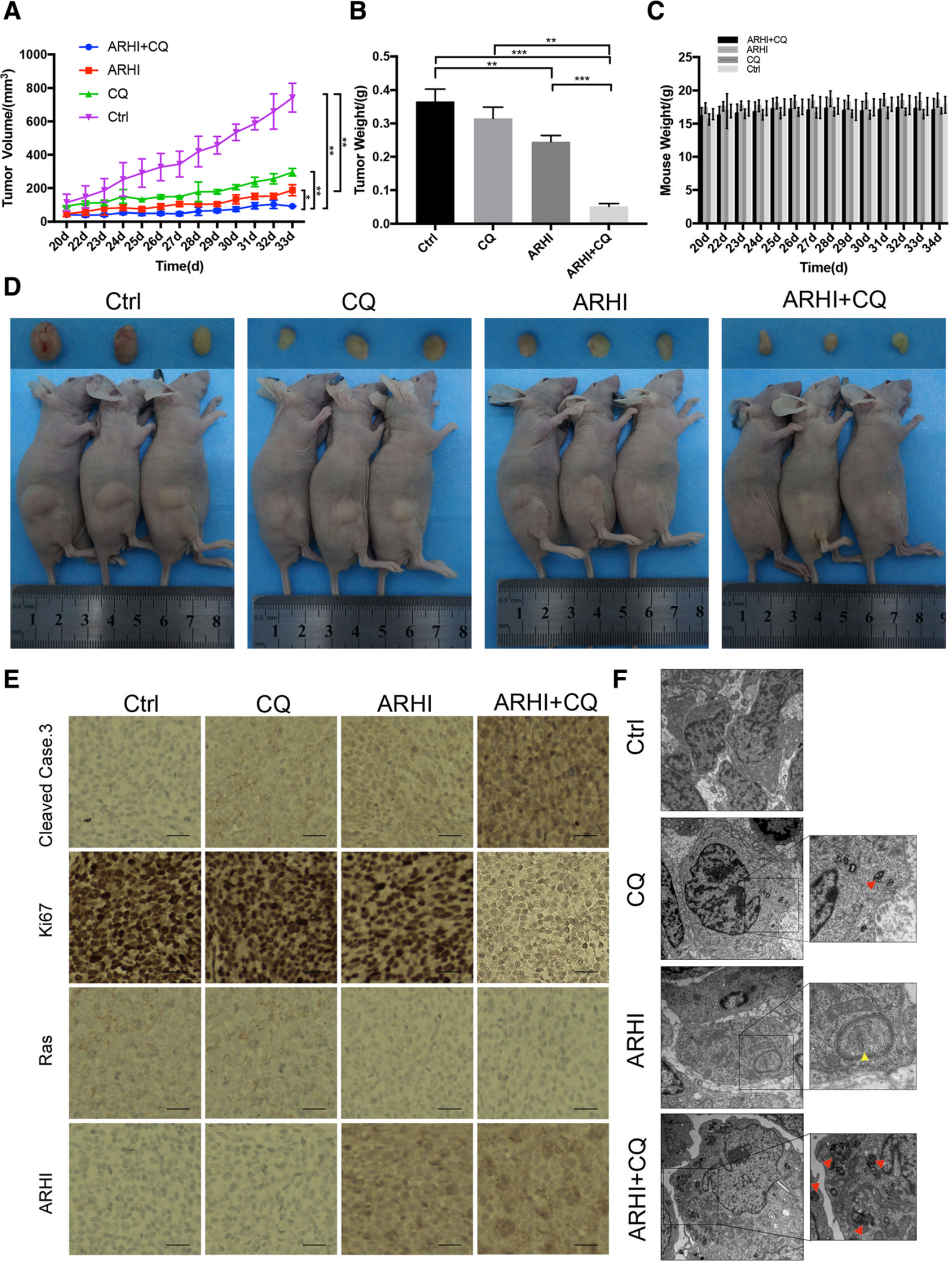
CQ enhances the cancer suppression of ARHI in vivo. **a** Tumor volume changes in the four xenograft models. **b** Tumor weight of the four groups after different treatment. **c** Body weight changes of mouse models. **d** Dissected tumors from four xenograft models. **e** Immunohistochemistry of cleaved caspase-3, Ki67, Ras and ARHI in vivo. The scale bar represents 50 μm. (the bar whiskers represent SD, *p < 0.05, **p < 0.01, ***p < 0.001). **f** TEM photomicrographs of the four xenograft models. AVs, autophagic vacuoles; AVi, initial AVs (yellow arrows); AVd, late AVs (red arrows). The scale bar represents 2 μm

Next, we assessed the efficacy of CQ-associated ARHI therapy in vivo. Either autophagosomes or apoptosis levels were increased after CQ-associated ARHI therapy and tumor growth was significantly suppressed. As shown in Fig. 6a, after CQ-associated ARHI treatment, the suppression of tumor growth was observed by gross inspection. By the way, body weight showed no significant difference among the four groups over four weeks (Fig. 6c). The therapeutic effect was assessed by tumor weight and volume. CQ-associated ARHI significantly inhibited tumor growth, which resulted in decreased tumor weight and volume in this group (Fig. 6b, d). In addition, elevated levels of cleaved caspase-3, LC3B and decreased levels of Ki67 and Ras were observed in the CQ-associated ARHI treatment group compared with the control group (Fig. 6e, Additional file 5). Taken together, these data support the hypothesis that CQ-associated ARHI exhibits anti-glioma properties in vivo. At last, we use TEM to detect autophagy markers. Compared with control, in ARHI group, more initial autophagy vacuoles (AVi) were observed in cytoplasm and in the group of CQ-associated ARHI, more AVd were accumulated in cytoplasm. All these indicated ARHI can also induce autophagy in vivo and that there were more autophagic vacuoles (almost all late autophagic vacuoles) accumulated in the CQ-associated group (Fig. 6f).

## Discussion

**Fig. 7:**
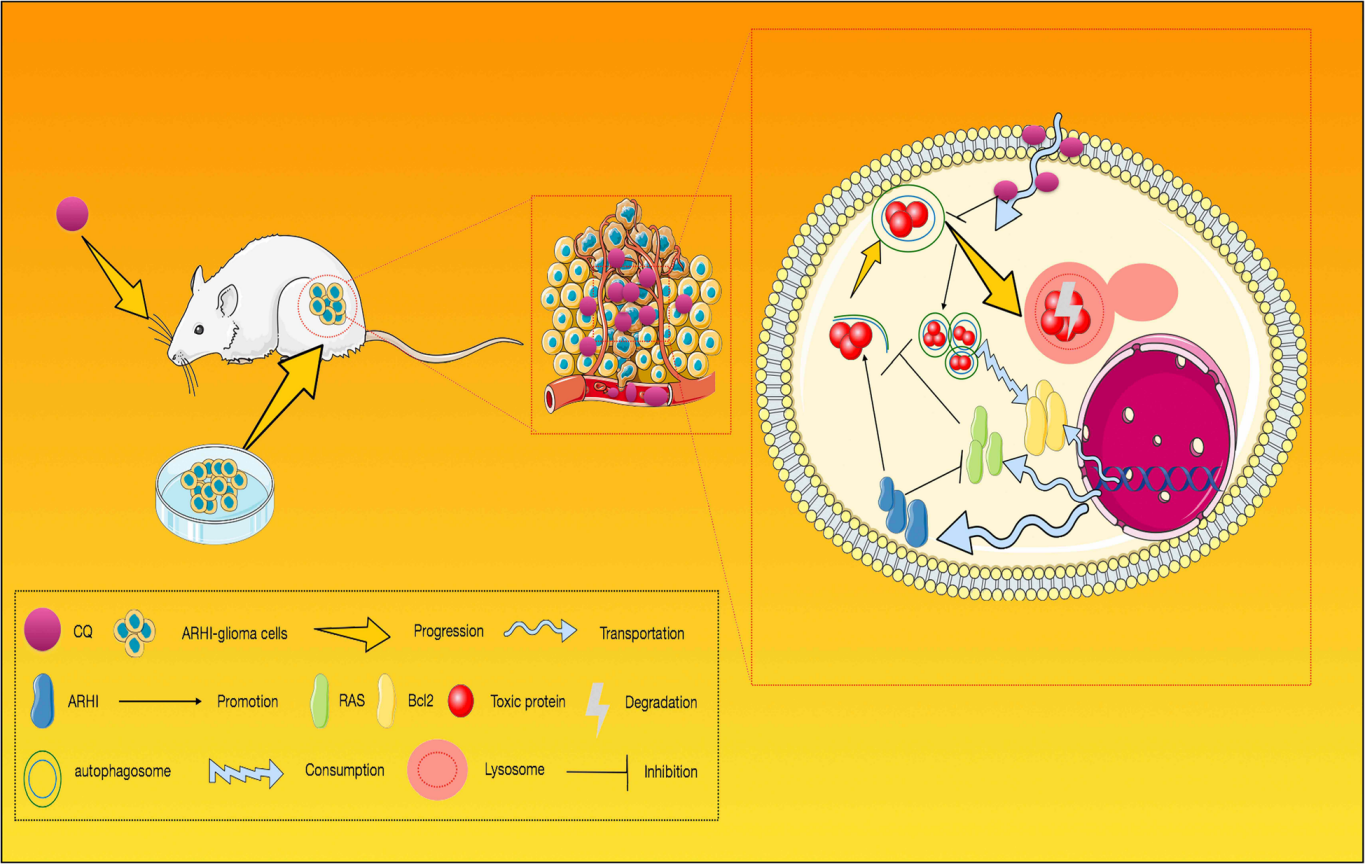
According to our research, we mapped the schematic model for the molecular mechanism of CQ in ARHI-glioma auxiliary chemotherapy

Glioma is the most common type of malignant brain tumor in adults. In which, glioblastoma multiforme (GBM) is the deadliest CNS tumor and is characterized by excessive proliferation, aggressive invasion and high resistance to conventional therapies [29, 30]. New therapeutic techniques are urgently needed, and gene therapy is an indispensable tool [31]. Tumor suppressor genes and oncogenes have long been used as therapeutic targets in multiple cancers [32, 33]. The appearance of tumors usually begins with the mutation of genes [34]. To understand cancer, we must first understand the oncogenes and anti-oncogenes [35]. Autophagy is a conserved process that captures, degrades, and recycles organelles, protein and other substances in cells, but excessive autophagy may induce cell death [36]. Our results, as shown in Fig. 7, suggested for the first that ARHI induced autophagy and apoptosis in GBM cells, and decreased the expression of Ki67 and colony formation in vitro and vivo. ARHI will be a potent anti-glioma agent after further research.

In our study, we found that ARHI was frequently downregulated in glioma tissues compared with normal brain tissues by qRT-PCR and western blot. Moreover, the expression level of ARHI was inversely associated with tumor grade. Furthermore, over-expressing ARHI can induce autophagy-mediated cell death in glioma, which further supports the hypothesis that ARHI represents a tumor suppressor gene in glioma. Previous studies have shown that ARHI gene expression products can inhibit the expression of Ras and ERK which is the downstream of Ras. Further studies found that the expression of ARHI decreased both PI3K and Ras/MAP signaling by downregulating EGFR through enhanced internalization and degradation leading to a shortened half-life, and decreased mTOR activity initiates autophagy. Mammalian target of rapamycin (mTOR) signaling pathway play a direct role in regulating autophagy [37]. Decreased p-AKT can inhibit mTOR activity through the AKT/mTOR pathway. According to our study, over-expression of ARHI can decrease the expression of Ras in glioma cells, thus indirectly suppressing mTOR activity and ultimately initiating autophagy. Moreover, ARHI can inhibit GBM cells proliferation and decrease tumorigenicity through inducing autophagic death. CQ can promote this effect through increasing AVd, enhancing the accumulation of cytotoxic protein and reducing anti-apoptotic protein Bcl-2, suggesting that ARHI may be a potential therapeutic target for glioma. Autophagosome formation has been implicated in the process of apoptosis [38, 39], and inhibition of the early stagy of autophagy reduced the activation of caspase-3, while the effect of the late stage of autophagy inhibition was opposite [40–42].

## Conclusions

In our study, we found that both autophagy and apoptosis were induced after over-expressing ARHI in glioma cells. Inhibiting autophagy by 3-MA led to decreased apoptosis and lower levels of the apoptosis marker cleaved caspased-3, indicating that inhibition of the early stage of autophagy reduced caspase-dependent apoptosis. CQ, as an inhibitor of late autophagy, combined with ARHI can enhance the accumulation of autophagic vacuoles and indirectly enhance the accumulation of cytotoxic protein and promoting apoptosis in glioma cells (Fig. 7). Thus, to sum up, ARHI may be a functional tumor suppressor in glioma. And CQ used as an auxiliary medicine in glioma chemotherapy can enhance the antitumor effect of ARHI, and this study provides a novel mechanistic basis and strategy for glioma therapy.

## Additional files

Additional file 1: Patients’ information. The table of patients’ information used in this research. (PDF 16 kb)

Additional file 2: The ARHI expression level in patients’ pathological tissues. Immunohistochemistry of ARHI in patients’ pathological tissues. The scale bar represents 200 μm. (JPG 6565 kb)

Additional file 3: ARHI over-expresssion can inhibit proliferation of glioma stem cells. (a) Fluorescence microscopy of glioma stem cell marker CD133 and SOX2 expression after transfection with ARHI and negative control plasmids. The scale bar represents 100 μm. G#1: patient1-derived cells. (b) Phase contrast microscopy of glioma stem cells inhibited by ARHI. Scale bar represents 100 μm. (c) ARHI mRNA relative expression in NHA cells、patient-derived cells and glioma stem cells. (JPG 1040 kb)

Additional file 4: ARHI over-expression can induce autophagy and inhibit proliferation in patient-derived primary cells. (a) Fluorescence microscopy of LC3B expression after transfection with ARHI and negative control plasmids. The scale bar represents 100 μm. (G#2: patient2-derived cells) (b) After transfecting with ARHI and negative control plasmids for 72 h, the expression levels of ARHI, SQSTM1,LC3B,Ras,phosphorylated and total mTOR and AKT were assessed by western blotting. Using total mTOR and AKT as the internal controls, the expression levels of phosphorylated mTOR and AKT were calculated (G#3: patient3-derived cells, the bar whiskers represent SD, *p < 0.05, **p < 0.01, ***p < 0.001). (c) Cell viability of patient3-derived cells after over-expressing ARHI or inhibiting autophagy by shATG5 (G#3: patient3-derived cells,the bar whiskers represent SD, *p < 0.05, **p < 0.01, ***p < 0.001). (d) The intracranial tumor size in orthotopic xenograft model. Scale bar represent 2000 μm. The survival time of nude mice bearing glioma. (G#1: patient1-derived cells) (JPG 1224 kb)

Additional file 5: ARHI can induce autophagy in vivo. (a) Immunohistochemistry of SQSTM1, LC3, and Bcl2 in vivo. The scale bar represents 50 μm. (b) The expression levels of SQSTM1, LC3, Ras, ARHI, phosphorylated and total mTOR, AKT were assessed by western blotting. Using total mTOR and AKT as the internal controls, the expression levels of phosphorylated mTOR and AKT were calculated (the bar whiskers represent SD, *p < 0.05, **p < 0.01, ***p < 0.001). (JPG 1819 kb)

## Abbreviations

3-MA: 3-methyladenine
ANT: adjacent normal tissue
ARHI: Aplasia Ras homolog member I
AVd: late or degradative autophagic vacuoles
AVi: initial autophagic vacuoles
AVs: autophagic vacuoles
CNS: central nervous system
CQ: chloroquine
GBM: glioblastoma multiforme
TEM: transmission electron microscopy
