# Supplementary material for: Oncogenic Ras is downregulated by ARHI and induces autophagy by Ras/AKT/mTOR pathway in glioblastoma
Source: BMC Cancer. 2019 May 14;19:441. doi: 10.1186/s12885-019-5643-z (PMC6515631; doi:10.1186/s12885-019-5643-z)
Supplement: Supplementary file 1 — Patients’ information. The table of patients’ information used in this research. (PDF 16 kb) [file 12885_2019_5643_MOESM1_ESM.pdf]

Features pathological diagnoses	WHO II	WHO III	WHO IV
No. of patient	3	3	3
Mean age (year)	43.3	51.6	45.6
Gender			
Male	2	1	1
Female	1	2	2
Tumor location			
Supratentorial	3	3	3
Tumor size(cm)			
<3	1	0	1
>3	2	3	2
KPS			
>80	2	2	3
<80	1	1	0
Alive	2	1	0
Average survival data(month)	49.5	32.7	10.2
