# Supplementary figures and images for: Oncogenic Ras is downregulated by ARHI and induces autophagy by Ras/AKT/mTOR pathway in glioblastoma
Source: BMC Cancer. 2019 May 14;19:441. doi: 10.1186/s12885-019-5643-z (PMC6515631; doi:10.1186/s12885-019-5643-z)

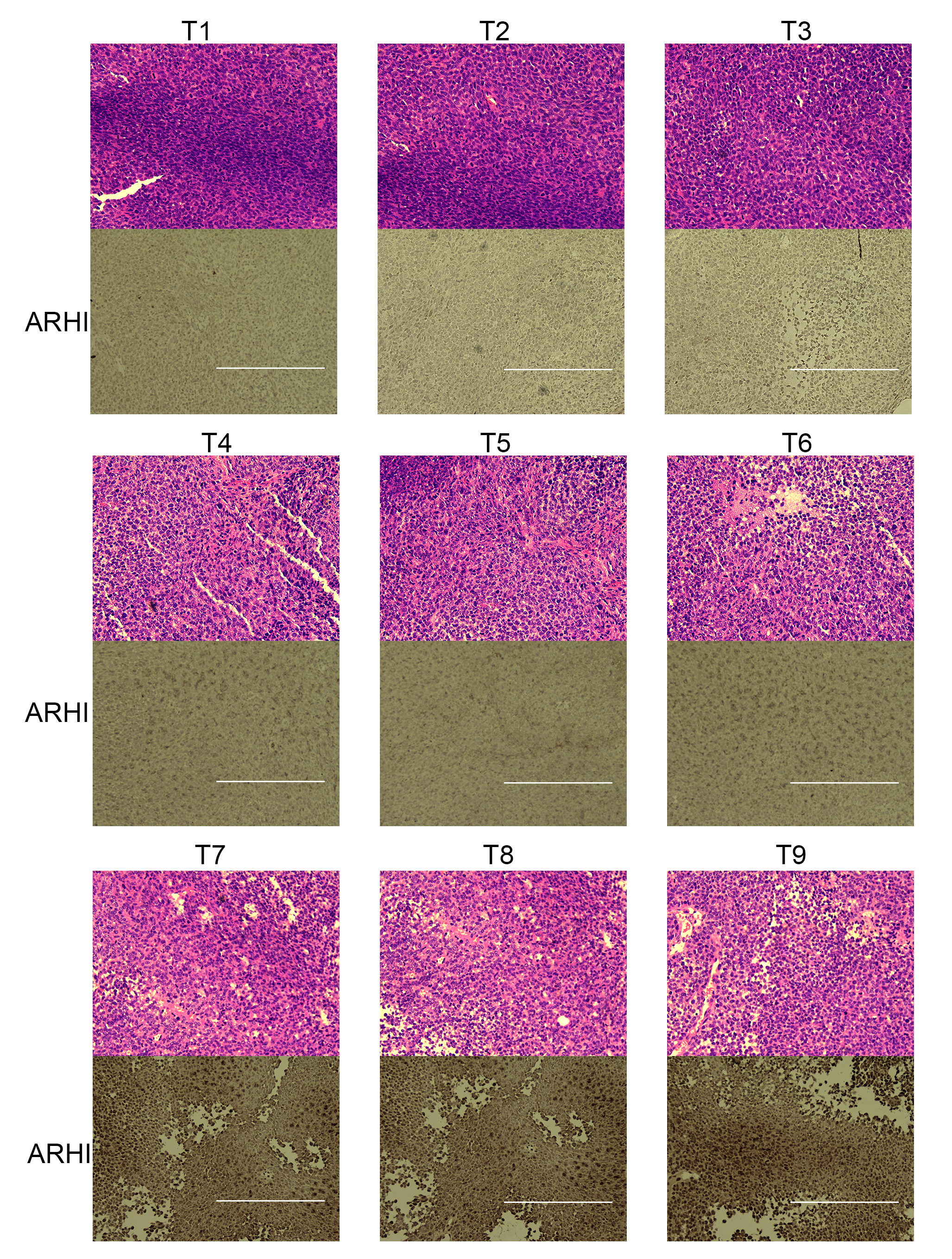

Supplement: Supplementary file 2 — The ARHI expression level in patients’ pathological tissues. Immunohistochemistry of ARHI in patients’ pathological tissues. The scale bar represents 200 μm. (JPG 6565 kb) [file 12885_2019_5643_MOESM2_ESM.jpg]

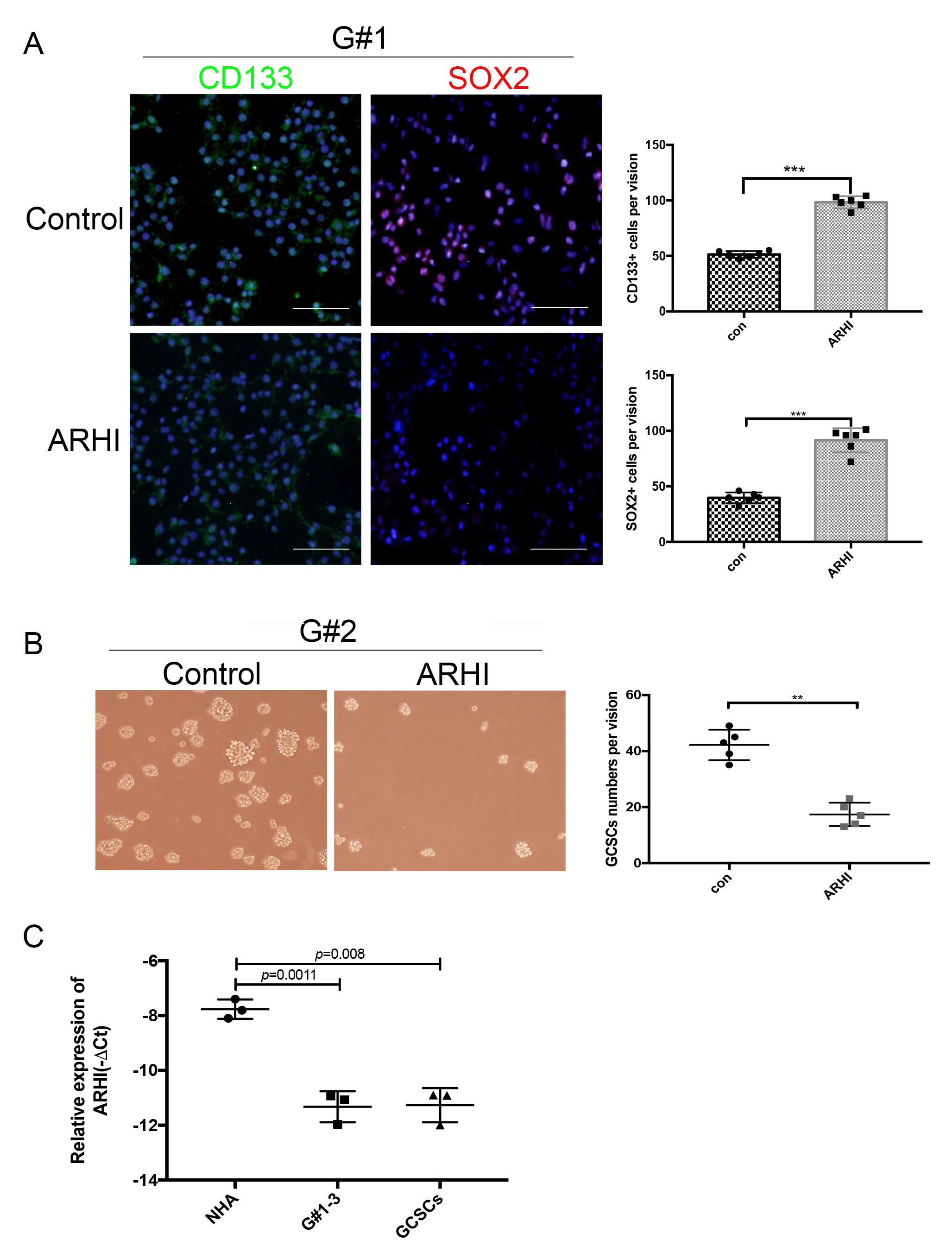

Supplement: Supplementary file 3 — ARHI over-expresssion can inhibit proliferation of glioma stem cells. (a) Fluorescence microscopy of glioma stem cell marker CD133 and SOX2 expression after transfection with ARHI and negative control plasmids. The scale bar represents 100 μm. G#1: patient1-derived cells. (b) Phase contrast microscopy of glioma stem cells inhibited by ARHI. Scale bar represents 100 μm. (c) ARHI mRNA relative expression in NHA cells、patient-derived cells and glioma stem cells. (JPG 1040 kb) [file 12885_2019_5643_MOESM3_ESM.jpg]

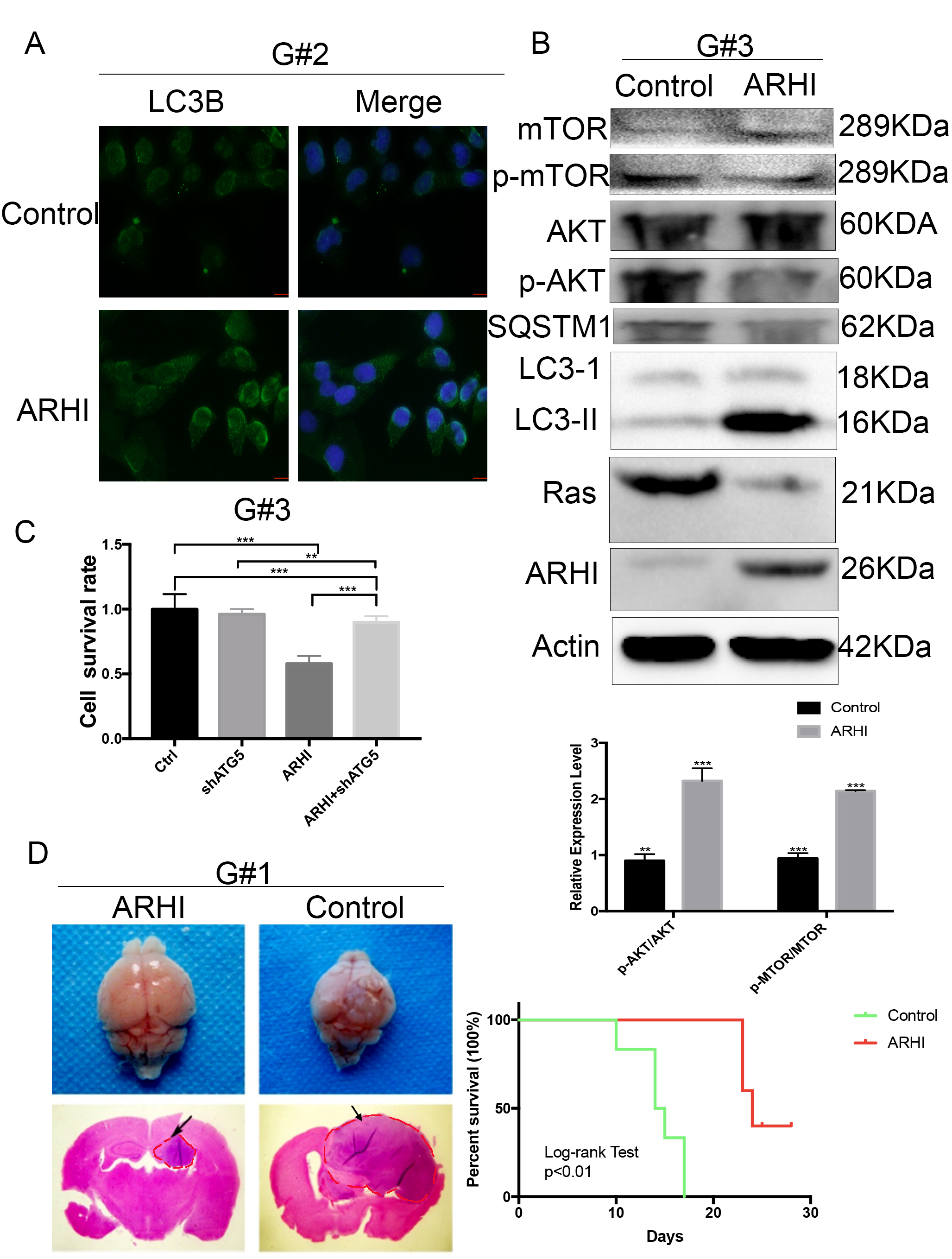

Supplement: Supplementary file 4 — ARHI over-expression can induce autophagy and inhibit proliferation in patient-derived primary cells. (a) Fluorescence microscopy of LC3B expression after transfection with ARHI and negative control plasmids. The scale bar represents 100 μm. (G#2: patient2-derived cells) (b) After transfecting with ARHI and negative control plasmids for 72 h, the expression levels of ARHI, SQSTM1,LC3B,Ras,phosphorylated and total mTOR and AKT were assessed by western blotting. Using total mTOR and AKT as the internal controls, the expression levels of phosphorylated mTOR and AKT were calculated (G#3: patient3-derived cells, the bar whiskers represent SD, *p < 0.05, **p < 0.01, ***p < 0.001). (c) Cell viability of patient3-derived cells after over-expressing ARHI or inhibiting autophagy by shATG5 (G#3: patient3-derived cells,the bar whiskers represent SD, *p < 0.05, **p < 0.01, ***p < 0.001). (d) The intracranial tumor size in orthotopic xenograft model. Scale bar represent 2000 μm. The survival time of nude mice bearing glioma. (G#1: patient1-derived cells) (JPG 1224 kb) [file 12885_2019_5643_MOESM4_ESM.jpg]

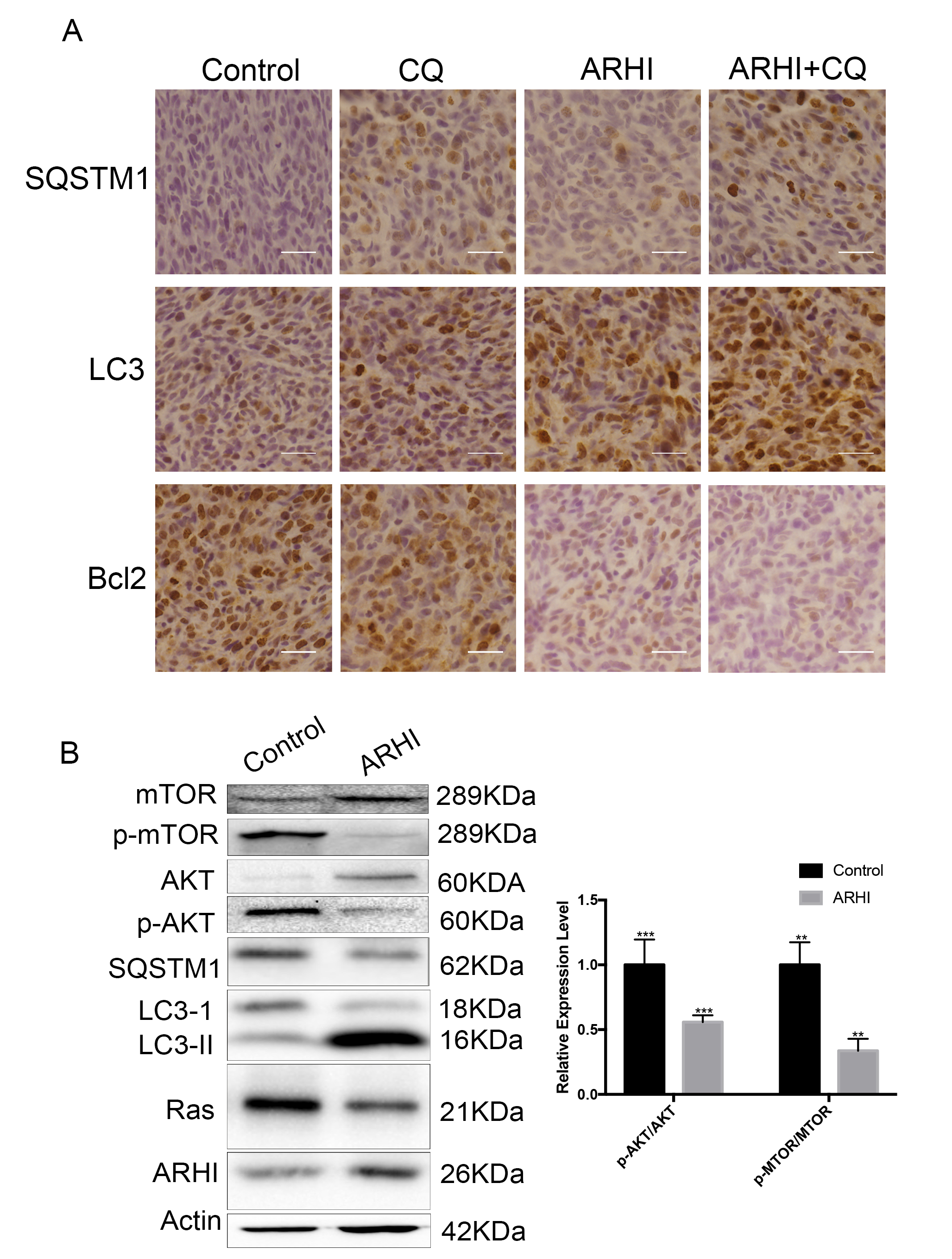

Supplement: Supplementary file 5 — ARHI can induce autophagy in vivo. (a) Immunohistochemistry of SQSTM1, LC3, and Bcl2 in vivo. The scale bar represents 50 μm. (b) The expression levels of SQSTM1, LC3, Ras, ARHI, phosphorylated and total mTOR, AKT were assessed by western blotting. Using total mTOR and AKT as the internal controls, the expression levels of phosphorylated mTOR and AKT were calculated (the bar whiskers represent SD, *p < 0.05, **p < 0.01, ***p < 0.001). (JPG 1819 kb) [file 12885_2019_5643_MOESM5_ESM.jpg]
